# Ethnic and Gender Disparities in Risk Factors for Prediabetes—A Retrospective Exploratory Analysis in Southern Israel

**DOI:** 10.3390/jcm15134893

**Published:** 2026-06-23

**Authors:** Michael Murninkas, Daniel Ostrovsky, Aya Biderman, Idit F. Liberty

**Affiliations:** 1 Clinical Research Center, Soroka University Medical Center, Ben-Gurion University of the Negev, Beer-Sheva 84105, Israel; michamur26@gmail.com (M.M.);; 2 Department of Internal Medicine B, Sheba Medical Center, Tel Hashomer, Ramat Gan 52621, Israel; 3 Department of Family Medicine and Siaal Research Center for Family Medicine and Primary Care, The Haim Doron Division of Community Health, Faculty of Health Sciences, Ben-Gurion University of the Negev, Beer-Sheva 84105, Israel; 4 Research Unit of Clalit Health Services, Southern District, Beer-Sheva 84260, Israel; 5 Diabetes Clinic, Soroka University Medical Center, Faculty of Health Sciences, Ben-Gurion University of the Negev, Beer-Sheva 84105, Israel; idit.liberty@gmail.com

**Keywords:** prediabetes, risk factors, ethnicity, gender, metabolic syndrome

## Abstract

**Background/Objectives:** Prediabetes significantly increases the risk of type 2 diabetes and related complications. Limited data exist for prediabetes among minority groups in Israel, particularly Bedouins. In the Negev region, Jewish and Bedouin populations differ markedly in culture and socioeconomic status. This study aimed to identify gender- and ethnicity-specific predictors of prediabetes. **Methods:** This retrospective, population-based observational exploratory study used data from 28,754 adults aged 20–65 years insured by Clalit Health Services in Southern Israel (2010–2020). Individuals with prediabetes were matched 1:1 with controls by age, gender, ethnicity, and year of diagnosis. Multivariate logistic regression models stratified by gender and ethnicity identified independent predictors. **Results:** Prediabetes was identified at significantly younger ages among Bedouins than Jews (6.8 years in men, 11.3 in women). The strongest predictor across all subgroups was metabolic syndrome (OR 2.0–4.0). Gestational diabetes was a major risk factor in women, particularly Jewish (OR 3.6). Cardiovascular disease and the use of statins or thiazide diuretics were independently associated with increased odds of prediabetes. Triglyceride-to-HDL cholesterol ratio was consistently elevated among prediabetes patients. **Conclusions:** Metabolic and medication-related factors contribute significantly to prediabetes-associated risk, with distinct gender and ethnic patterns. Culturally tailored early interventions and individualized risk profiling may enhance diabetes prevention in Southern Israel.

## 1. Introduction

Prediabetes represents a spectrum of abnormal glucose metabolism that does not yet meet the diagnostic criteria for type 2 diabetes. According to the American Diabetes Association (ADA), it can be defined by either impaired fasting glucose (IFG: fasting glucose 100–125 mg/dL), impaired glucose tolerance (IGT: 2-h plasma glucose 140–199 mg/dL during a 75-g oral glucose tolerance test [OGTT]), or HbA1c levels of 5.7–6.4% [1].

In 2021, the global prevalence of IGT was estimated at 9.1% (464 million) and is projected to rise to 10.0% (638 million) in 2045. Similarly, the prevalence of IFG was 5.8% (298 million), expected to increase to 6.5% (414 million) by 2045. While the highest prevalence is currently found in high-income countries, the steepest increases are anticipated in low-income countries [2].

Prediabetes substantially increases the risk of progression to type 2 diabetes [3]. Moreover, affected individuals face elevated risks for similar complications as type 2 diabetes, such as cardiovascular diseases, renal impairment, certain cancers, accelerated cognitive decline, and reduced life expectancy [4,5,6,7,8,9,10,11].

From a preventive medicine perspective, a diagnosis of prediabetes often represents a relatively late stage in the pathophysiological continuum toward type 2 diabetes. Current prevention strategies primarily focus on individuals with prediabetes, employing lifestyle interventions and, in some cases, pharmacologic therapy to delay or prevent the onset of type 2 diabetes and its associated complications [12,13].

Ethnic disparities in the prevalence of prediabetes and type 2 diabetes have been well documented in different communities, particularly among underserved populations, such as Mexican Americans [14], USA children and adolescents [15], and ethnically diverse adults in the United States [16].

In Israel, higher rates of type 2 diabetes have been observed among certain minority groups, including Muslim population [17,18]. However, limited data exist regarding the prevalence of prediabetes in these groups. Recent Israeli epidemiological studies demonstrated higher diabetes and metabolic risk among Arab populations compared with Jewish populations [19].

In the Negev region of southern Israel, two principal ethnic populations coexist: Jewish and Bedouin. These groups differ substantially in sociodemographic and cultural characteristics. The Bedouin population is currently transitioning from a traditionally nomadic and physically active lifestyle to more sedentary, Westernized behaviors, contributing to rising rates of obesity, metabolic syndrome, and diabetes mellitus. Compared to the Jewish population, Bedouins tend to be younger, exhibit higher fertility rates, lower educational attainment and socioeconomic status, and higher unemployment [20,21]. Approximately 77% reside in recognized townships, while the rest live in unrecognized villages [20].

According to the Israel Central Bureau of Statistics and the Arab Society Statistical Report 2023, Bedouin citizens comprise roughly one-third of the Negev population [22].

Clalit Health Services (CHS), the largest health maintenance organization (HMO) in Israel, insures approximately 70% of the Southern District’s population. Its comprehensive, fully computerized electronic medical records (available since 2000) provide a valuable platform for population-based research.

While age, sex and BMI are consistent predictors for prediabetes across previous studies, additional risk factors vary by region and population. In Qatar, for example, waist circumference and blood pressure were key predictors [23]. The Indonesian Prediabetes Risk Score (INA-PRISC) included education level, family history, smoking, physical activity, BMI and hypertension [24]. U.S National Health and Nutrition Examination Survey (NHANES) data from 1999–2004 identified age, sex, BMI, family history, resting heart rate, and hypertension to be stronger predictors than BMI alone [25]. The diabetic risk score was developed to predict drug-treated diabetes in Finland. The study used age, BMI, waist circumference, history of antihypertensive drug treatment and high blood glucose, physical activity, and daily consumption of fruits, berries, or vegetables as categorical variables. The score did not address the risk for pre-diabetes [26].

Given the unique demographic, socioeconomic, and cultural differences between the Jewish and Bedouin populations in the Negev, and the higher prevalence of type 2 diabetes among Bedouins, this study aimed to identify specific predictors of prediabetes to support more targeted and effective prevention strategies.

## 2. Methods

### 2.1. Data Source and Ethical Approval

We conducted a retrospective, population-based, observational exploratory study in the Southern district of Israel between 2010 and 2020, based on the Clalit Health Services electronic medical records database, one of the largest and most comprehensive health maintenance organization (HMO) databases worldwide, via the MDClone-powered platform [27]. Eligible participants included adults aged 20–65 years from the Jewish and Bedouin populations. This age range was chosen to capture adults likely to undergo routine health screenings while avoiding confounding effects related to elderly comorbidities.

Prediabetes cases were defined based on two laboratory tests within two years, demonstrating fasting glucose levels of 100–125 mg/dL or HbA1C levels of 5.7–6.4%. OGTT data were not systematically available in the database; therefore, isolated impaired glucose tolerance (IGT) cases may not have been identified. Controls were matched 1:1 to cases using nearest-neighbor matching by age (within two years), gender, ethnicity, and the year of diagnosis or follow-up date. An inclusion criterion for controls was having glucose level tests performed within five years prior to follow-up.

Exclusion criteria included any history of fasting glucose above 125 mg/dL, HbA1c above 6.4%, prior Diabetes Mellitus diagnosis, or prior metformin or insulin prescriptions. After exclusions, 28,754 participants were included, comprising 14,377 individuals with prediabetes and 14,377 matched controls.

Collected variables included demographics (age, gender, ethnicity, BMI), chronic diseases diagnoses (including ischemic heart disease [IHD], hypertension [HTN], dyslipidemia, peripheral vascular disease [PVD], gestational diabetes mellitus [GDM], cerebrovascular accident [CVA], and transient ischemic attack [TIA]), and medication use (acetylsalicylic acid [ASA], statins [STA] and thiazide diuretics [TZD]).

Obesity was defined categorically as a BMI above 30 kg/m^2^. Cardiovascular disease (CVD) included any history of IHD, PVD, CVA, or TIA. Metabolic syndrome components were approximated according to the 2009 Harmonized criteria using available database variables, including hypertension, obesity (BMI > 30 kg/m^2^), triglycerides > 150 mg/dL, and HDL cholesterol < 40 mg/dL in men or <50 mg/dL in women.

### 2.2. Statistical Analysis

Descriptive statistics were used to summarize continuous variables as means ± standard deviations (SDs) and categorical variables as percentages. Univariate analyses employed chi-square tests for categorical variables and *t*-tests for continuous variables. The population was stratified into four subgroups by sex and ethnicity to account for observed differences.

Multivariate logistic regression models were used to identify independent predictors of prediabetes. Potential multicollinearity among variables was assessed using Pearson correlation matrices and clinical judgment during model construction. Variables were selected based on clinical relevance and strength of association in the univariate analysis and entered in a stepwise forward selection approach. The final multivariable logistic regression models included metabolic syndrome, cardiovascular disease, gestational diabetes mellitus, statin use, and thiazide diuretic use. Definitions of metabolic syndrome and cardiovascular disease are provided above. Analyses were conducted using RStudio version 2022.07.1 (Posit Software, PBC, Boston, MA, USA), with *p*-values < 0.05 considered statistically significant. Internal validation was performed using bootstrap resampling with 500 repetitions of the multivariable logistic regression models within each subgroup.

## 3. Results

### 3.1. Baseline Characteristics

Participants were equally distributed between prediabetes and control groups, matched by age, gender and ethnicity. Jewish participants were significantly older than Bedouins (Jewish males: 45.6 ± 12.7 vs. Bedouin males: 39.28 ± 10.88, Jewish females: 48.33 ± 12.12 vs. Bedouin females: 37.4 ± 11.7 years) (Table 1 and Table 2). This illustrates a striking average difference in age of diagnosis for prediabetes of 6.8 years in males and 11.3 years in females.

### 3.2. Clinical Risk Factors and Laboratory Tests

Individuals with prediabetes exhibited a higher prevalence of metabolic risk factors across all subgroups compared to controls. In Jewish males, prediabetes was associated with significantly higher rates of obesity (29.7% vs. 13.3%, *p* < 0.001), hypertension (24.1% vs. 12.6%, *p* < 0.001), cardiovascular disease (8.9% vs. 4.3%, *p* < 0.001), and metabolic syndrome (14.9% vs. 4.6%, *p* < 0.001). Jewish females with prediabetes exhibited significantly higher prevalence of obesity (39.4% vs. 15.3%, *p* < 0.001), hypertension (25.4% vs. 13.1%, *p* < 0.001), cardiovascular disease (5.2% vs. 3.1%, *p* < 0.001), metabolic syndrome (16.6% vs. 4.3%, *p* < 0.001), and gestational diabetes (1.7% vs. 0.5%, *p* < 0.001). Among Bedouin males, prediabetes was associated with obesity (24.1% vs. 12.1%, *p* < 0.001), hypertension (11.8% vs. 6.2%, *p* < 0.001), cardiovascular disease (4.5% vs. 1.5%, *p* < 0.001), and metabolic syndrome (13.4% vs. 6.7%, *p* < 0.001). Bedouin females demonstrated similar patterns, with higher rates of obesity (42.8% vs. 21.6%, *p* < 0.001), hypertension (13.3% vs. 7.9%, *p* < 0.001), cardiovascular disease (1.7% vs. 0.8%, *p* < 0.001), metabolic syndrome (16.9% vs. 7.2%, *p* < 0.001), and gestational diabetes (4.6% vs. 2.6%, *p* < 0.001).

The triglyceride to HDL cholesterol ratio (TG/HDL) was notably higher in the prediabetes groups across all subgroups. This ratio, a marker of insulin resistance and dyslipidemia, was significantly elevated among Jewish males (4.12 ± 4.06 vs. 3.07 ± 2.32, *p* < 0.001), Jewish females (2.65 ± 1.8 vs. 1.9 ± 1.2, *p* < 0.001), Bedouin males (4.58 ± 4.01 vs. 3.71 ± 2.51, *p* < 0.001), and Bedouin females (2.73 ± 1.87 vs. 2.16 ± 1.43, *p* < 0.001). These findings are consistent with previous studies highlighting the TG/HDL ratio as a strong predictor of prediabetes and progression to type 2 diabetes [28].

As expected, fasting glucose levels were significantly higher across all prediabetes groups as per inclusion criteria. Serum creatinine was slightly but significantly elevated among prediabetes patients in most groups. Statin and thiazide diuretic use were significantly more prevalent among prediabetes patients across all subgroups. For example, statin use among Jewish females with prediabetes was 25.7% vs. 14.2% in controls (*p* < 0.001). (Table 1 and Table 2). Unfortunately, we did not have access to the type of statin used, although we acknowledge that the risk of developing diabetes varies depending on the type of statin used [29].

### 3.3. Multivariate Logistic Regression Analysis

**Figure 1:**
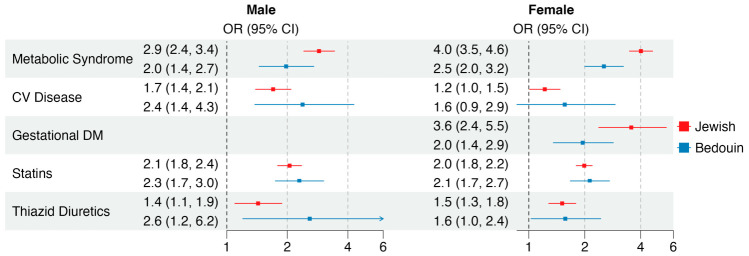
Multivariate logistic regression analysis displaying independent predictors of prediabetes stratified by gender and ethnicity.

The results of the final multivariable logistic regression models are presented in Table 3 and Figure 1. In the multivariable logistic regression analysis, metabolic syndrome remained the most robust predictor of prediabetes across all groups. Among Jewish males the odds ratio (OR) for metabolic syndrome was 2.9 (95% CI 2.4–3.4), for Jewish females 4.0 (95% CI 3.5–4.6), Bedouin males 2.0 (95% CI 1.4–2.7), Bedouin females 2.5 (95% CI 2.0–3.2). Gestational diabetes mellitus (GDM) was strongly associated with prediabetes in both Jewish females (OR 3.6; 95% CI 2.4–5.5) and Bedouin females (OR 2.0; 95% CI 1.4–2.9). Figure 1 summarizes the adjusted odds ratios derived from the multivariate logistic regression models. This highlights GDM’s significant role as a predictor of prediabetes and its importance in female-specific risk profiles. Cardiovascular disease (CVD) was a notable risk factor particularly in males. Among Jewish males, the OR for CVD was 1.7 (95% CI: 1.4–2.1), and among Bedouin males, it was even higher- 2.3 (95%CI: 1.3–4.2). However, in females, CVD was a significant predictor only in the Jewish group (OR 1.2, 95% CI 1.0–1.5), indicating a striking gender disparity in its association with prediabetes.

The use of statins and thiazide diuretics was a significant risk factor across all four groups, consistently associated with increased odds of prediabetes.

Overall, the multivariate analysis emphasizes the central role of metabolic syndrome, CVD, GDM, and use of statins and thiazide diuretics as key predictors. Internal validation using bootstrap resampling (500 repetitions) demonstrated highly similar confidence intervals to the original estimates, supporting the stability and robustness of the multivariable models (Supplementary Table S1).

The model includes variables such as metabolic syndrome (defined as having ≥ 3 components), hypertension (HTN), dyslipidemia, obesity (BMI > 30 kg/m^2^), ischemic heart disease (IHD), peripheral vascular disease (PVD), cerebrovascular accident (CVA), transient ischemic attack (TIA), gestational diabetes mellitus (GDM), statin use, and thiazide diuretic use. Metabolic syndrome was consistently the strongest predictor across groups. GDM was a particularly strong predictor among females, especially in the Jewish subgroup.

## 4. Discussion

The primary aim of this study was to identify gender and ethnicity-specific risk factors for developing prediabetes among Jewish and Bedouin adults in Southern Israel. We observed a notable difference in the age of prediabetes diagnosis between the two ethnic groups, with Bedouin males being diagnosed 6.8 years earlier than Jewish males and Bedouin females 11.3 years earlier than Jewish females. This important finding was also described in earlier studies in Israel, regarding the predisposition to diabetes among Arabs and Jews [18].

Earlier diabetes diagnosis in minority populations compared to majority populations is a well-documented global phenomenon. In the US Asian, Black, and Hispanic individuals developed prediabetes at younger ages than White individuals [16].

Similar finding are shown in Europe South Asian populations show notably elevated T2DM prevalence, followed by Middle Eastern and North African populations, compared to host European populations and in Australia with the Aboriginal and Torres Strait Islander [30]. The mechanisms underlying earlier prediabetes and diabetes onset in minority populations share similarities with the Bedouin experience: higher obesity rates [19], biological risk factors including differences in BMI, insulin resistance, and metabolic parameters account for much of the disparity, particularly among women [31]. Socioeconomic factors: lower education and income levels consistently associate with earlier diabetes onset across minority populations and was shown in the Bedouin population too [32,33]. Moreover, high rates of physical inactivity characterize the Bedouin and broader Arab population in Israel, with approximately 85% of men and 97% of women with diabetes being physically inactive [33].

On top of the age differences, ethnic dissimilarity in glycemic indices have also been demonstrated in Southern Israel, where HbA1c levels differed significantly between ethnic groups even among patients with established diabetes [34]. These observations further support the presence of substantial ethnic disparities in diabetes susceptibility within the Israeli population [19].

This earlier age at diagnosis in Bedouins carries important clinical implications. Along with younger age, Bedouin prediabetic participants exhibited lower rates of hypertension, dyslipidemia, and cardiovascular disease compared to Jewish participants, likely reflecting their shorter cumulative exposure to metabolic risk factors.

Across all subgroups, metabolic syndrome and its components emerged as the main risk factors for prediabetes, reaffirming findings from previous international studies [23,24,25,35,36]. Obesity was the strongest individual component, consistent with its central role in insulin resistance and glucose dysregulation globally [16,23,24] (Supplementary Figure S1). Interestingly, hypertension was also significantly associated with prediabetes in all groups except Bedouin females. This finding suggests that the relationship between blood pressure and glucose dysregulation may differ in Bedouin females. Rather than indicating a protective effect, the absence of a significant association may reflect differences in other cardiometabolic risk factors among Bedouin women, especially the prominent role of obesity in the development of glucose resistance. Previous studies have reported particularly high rates of obesity and diabetes in Arab and Bedouin women, identifying excess adiposity as a major contributor to impaired glucose metabolism [17,18]. In populations with a high burden of obesity, obesity-related metabolic abnormalities may have a stronger influence on prediabetes risk than hypertension alone, thereby attenuating the independent association between hypertension and prediabetes in multivariable analyses.

Mostly hypertension and prediabetes share common pathophysiological mechanisms centered on insulin resistance, sympathetic nervous system activation, and renin-angiotensin-aldosterone system (RAAS) dysregulation [37]. The absence of an association between hypertension and prediabetes in Bedouin women specifically is intriguing given documented sex differences in these relationships. Several factors may contribute: (1) a younger age distribution where estrogen protection remains robust; Women with DM have more hypertension at >60 y of age (i.e., postmenopausal) [38]. (2) Men accumulate more visceral fat, which is strongly linked to insulin resistance and hypertension, while women’s fat distribution patterns differ taken together with the younger age of the Bedouin women [39]. (3) population-specific genetic variants affecting insulin sensitivity or blood pressure regulation; or (4) lifestyle or dietary factors unique to this population that modify the relationship. 

Sociocultural and lifestyle factors may also contribute to this finding. Bedouin communities in southern Israel have undergone rapid urbanization and epidemiological transition over recent decades, accompanied by changes in dietary patterns, physical activity, and socioeconomic conditions [40]. These transitions have been linked to increasing rates of obesity, diabetes, and other metabolic disorders, particularly among women. Consequently, the pathways leading to prediabetes in Bedouin women may differ from those observed in other population groups, with obesity and lifestyle-related factors playing a more dominant role than hypertension [11,41].

Gestational diabetes mellitus (GDM) showed a strong association with prediabetes among women of both ethnicities. This finding echoes recent data from Germany [42], where over half of women with GDM developed prediabetes within five years postpartum, and about 6% had type 2 diabetes. Impaired fasting glucose (IFG) was the most common first indicator of disturbed glucose tolerance, followed by impaired glucose tolerance (IGT), the combination of IFG and IGT, and diabetes. Glucose tolerance did not deteriorate steadily in most women but fluctuated from year to year. Furthermore, a machine learning study [43] highlighted antenatal fasting glucose and HbA1c as early predictors of postpartum prediabetes in women with GDM, giving an area under the curve of 0.72, outperforming all other methods, and reinforcing the importance of early screening. In our study, GDM was more prevalent among Bedouin women, potentially due to higher parity. However, the stronger odds ratios observed among Jewish women may reflect differences in metabolic susceptibility or screening practices and highlight the need to explore whether postpartum follow-up and access to care differ between the two populations.

Notably, the triglyceride to HDL cholesterol (TG/HDL) ratio was significantly higher among prediabetic patients across all subgroups. This supports its use as a potential surrogate marker for insulin resistance and aligns with prior literature linking it to progression from prediabetes to diabetes [28]. The triglyceride/HDL ratio is also useful for identifying metabolic syndrome in patients with type 1 diabetes, making this index potentially useful in endocrine diseases [44]. Whether TG/HDL should be routinely incorporated into prediabetes risk scores in multiethnic settings is another important question emerging from our study.

Our study found that use of statins or thiazide diuretics were independently associated with increased odds of prediabetes. While the observational design limits causal inference, previous studies have shown that statins may increase the risk of type 2 diabetes, particularly among individuals with borderline glycemic indices [45,46].

Studies demonstrate that statins cause both decreased insulin sensitivity (24% reduction in one large observational study) and impaired insulin secretion (12% reduction) [47]. The key mechanistic pathway involves depletion of geranylgeranyl pyrophosphate (GGPP), an intermediate in the mevalonate pathway. GGPP is essential for protein geranylgeranylation, which is critical for glucose uptake in skeletal muscle. When statins block HMG-CoA reductase, GGPP levels fall, impairing glucose disposal and causing insulin resistance [48].

The risk of developing diabetes varies depending on the type of statin, where factors such as lipophilic or hydrophilic nature appear to have an impact. The risk is higher with rosuvastatin and lower with pravastatin [29]. Animal model studies have shown that high doses of rosuvastatin have the capacity to drastically modify mitochondrial morphology in hepatocytes, which could cause severe ATP depletion in cells [49]. Another mechanism proposed as a possible cause of the development of diabetes in some individuals treated with statins is cholesterol overload in pancreatic beta cells [50]. Animal models have demonstarted that atorvastatin can increase cholesterol transport in certain tissues, which could lead to cholesterol overload [51]. Unfortunately, detailed information regarding the specific statin brand and changes along the study period were not available in our database and should be evaluated in future studies.

Thiazide diuretics have been associated with impaired glucose metabolism [52], but a 2020 meta-analysis [53] suggests their impact on fasting glucose is not clinically significant.

One of the mechansisms described is through hypokalemia-induced reduction in insulin secretion being a primary mechanism. Thiazides open K-ATP channels in pancreatic β-cells, causing hyperpolarization that inhibits calcium influx and decreases insulin release [54]. Additional studies show inhibition of mitochondrial carbonic anhydrase 5b (CA5b) as a direct molecular mechanism. Thiazides inhibit CA5b in pancreatic β-cells, which is critical for replenishing oxaloacetate in the mitochondrial TCA cycle (anaplerosis). This inhibition attenuates Krebs cycle anaplerosis and reduces insulin secretion. Mice lacking CA5b are resistant to thiazide-induced glucose intolerance, confirming this pathway [55]. Thiazides also increase insulin resistance through activation of the renin-angiotensin system (RAS). Volume depletion reflexively increases RAS activity, and angiotensin II inhibits recruitment of insulin-sensitive adipocytes, causing redistribution of triglycerides to liver and skeletal muscle, promoting insulin resistance [54].

Several tools have been used for predicting type 2 diabetes: The Finnish Diabetes Risk Score (FINDRISC), originally developed and validated in Finland, is a widely used non-invasive tool to predict future risk of type 2 diabetes. This questionnaire includes eight questions/items based on self-disclosure, including age, BMI, waist circumference, physical activity, dietary habits, high blood pressure, history of high blood glucose, and family history of type 2 diabetes. With a score of 0–26, the FINDRISC has demonstrated good predictive performance for type 2 diabetes mellitus, with a sensitivity of 78%, a specificity of 77%, and a predictive value of a negative test of 99% [26,56]. This supports the external validity of our findings and highlights the utility of incorporating such validated risk scores, along with behavioral and familial factors, into future predictive models.

Our study’s strengths include the large, diverse sample size, drawn from comprehensive and high-quality electronic health records with validated laboratory and medication data. The meticulous matching strategy minimized confounding and allowed detailed gender and ethnicity-specific analyses. Universal healthcare access in Israel reduces socioeconomic disparities and strengthens the generalizability of our findings within the region.

A significant limitation of the study is the lack of analysis of variables related to lifestyle, diet, family history and possible other factors. All of these variables, which are known to influence prediabetes risk, could affect the differences found between the groups in this study [57,58]. These factors were not available in the electronic medical records and their absence may result in residual confounding.

Because OGTT measurements were not systematically available, isolated IGT cases may have been underrepresented. Medication adherence could not be assessed directly, further complicating interpretation of drug-prediabetes associations. As an observational exploratory analysis, the present study should primarily be interpreted as hypothesis-generating and cannot establish causal relationships. As the inclusion criteria required glucose testing within 5 years, potential selection bias may exist. Additionally, the study population was limited to Southern Israel, which may affect generalizability to other populations. A prospective cohort design with standardized OGTT measurements, direct assessment of insulin resistance, and systematic collection of lifestyle and socioeconomic variables would enable more accurate phenotyping of disglycemia. Although pairwise correlation assessment was performed during model construction, additional multicollinearity diagnostics such as variance inflation factor analysis were not available in the present revision.

Altogether, our results generate several new questions. Should age thresholds for metabolic screening differ between Bedouin and Jewish adults, given the substantially earlier onset of pre-diabetes in Bedouins? What mechanisms explain the weaker association between hypertension and prediabetes in Bedouin women, and the stronger impact of GDM among Jewish women? To what extent can simple markers such as TG/HDL or composite scores such as FINDRISC be adapted to provide accurate risk stratification in this specific multicultural context? Addressing these questions will be important for designing effective, population-appropriate prevention programs. The findings in this study, especially the specific ethnic differences in predictors of Diabetes Mellitus, highlight the need for tailored prevention strategies that reflect gender and ethnic specific profiles and reinforce the importance of early, population-specific interventions. The emphasis of lifestyle modifications in exercise and dietary changes is a central element in preventive efforts. This is supported by long-term results from the Diabetes Prevention Program and its Outcomes Study (DPPOS), which demonstrate that intensive lifestyle changes and metformin treatment significantly delayed diabetes onset and increased diabetes-free survival in individuals with prediabetes over a 21-year follow-up period [28].

## 5. Conclusions

This study aiming to identify specific predictors of prediabetes among Jewish and Bedouin populations, highlights the central role of metabolic syndrome and its components, in both populations, particularly obesity. Metabolic syndrome was consistently the strongest predictor. GDM further underscores the need for gender and ethnicity tailored screening strategies including earlier screening among Bedouins and close follow up of women who experienced GDM. The study reinforced the risk of statin and thiazide diuretics use in these high-risk populations.

Future prospective studies integrating behavioral, genetic, and socioeconomic variables are warranted to refine prediction models and to inform precision prevention efforts across diverse populations.

## Figures and Tables

**Table 1:** Baseline clinical characteristics and risk factors among Jewish and Bedouin males with and without prediabetes.

	Jewish			Bedouin		
	Control *n* = 4056	Prediabetes *n* = 4056	*p*-Value	Control *n* = 1276	Prediabetes *n* = 1276	*p*-Value
**Clinical Factors**						
Age, years	45.59 (12.69)	46.10 (12.93)	0.073	39.28 (10.88)	39.30 (10.89)	0.972
Obesity, *n* (%)	485 (13.3)	1154 (29.7)	<0.001	143 (12.1)	296 (24.1)	<0.001
CV Disease, *n* (%)	174 (4.3)	360 (8.9)	<0.001	19 (1.5)	57 (4.5)	<0.001
Hypertension, *n* (%)	512 (12.6)	978 (24.1)	<0.001	79 (6.2)	151 (11.8)	<0.001
Metabolic Syndrome, *n* (%)	185 (5.4)	559 (14.9)	<0.001	66 (6.7)	144 (13.4)	<0.001
**Laboratory**						
Triglycerides, mg/dL	128.20 (75.38)	162.23 (118.29)	<0.001	141.07 (79.04)	169.93 (117.30)	<0.001
HDL, mg/dL	45.93 (10.53)	43.96 (10.27)	<0.001	41.47 (8.82)	40.65 (8.56)	0.029
Triglycerides/HDL	3.07 (2.32)	4.12 (4.06)	<0.001	3.71 (2.51)	4.58 (4.01)	<0.001
Glucose, mg/dL	86.22 (9.35)	104.94 (8.29)	<0.001	85.75 (9.62)	105.43 (9.12)	<0.001
Creatinine, mg/dL	0.90 (0.14)	0.93 (0.42)	<0.001	0.84 (0.13)	0.89 (0.50)	0.004
**Drugs**						
Statins, *n* (%)	480 (11.8)	932 (23.0)	<0.001	97 (7.6)	214 (16.8)	<0.001
Thiazide Diuretics, *n* (%)	95 (2.3)	192 (4.7)	<0.001	8 (0.6)	33 (2.6)	<0.001

Continuous variables are presented as mean (SD) unless otherwise stated; categorical variables as *n* (%).

**Table 2:** Baseline clinical characteristics and risk factors among Jewish and Bedouin females with and without prediabetes.

	Jewish			Bedouin		
	Control *n* = 6872	Prediabetes *n* = 6872	*p*-Value	Control *n* = 2173	Prediabetes *n* = 2173	*p*-Value
**Clinical Factors**						
Age, years	48.33 (12.12)	49.06 (12.45)	0.001	37.40 (11.67)	37.72 (12.01)	0.371
Obesity, *n* (%)	979 (15.3)	2643 (39.4)	<0.001	444 (21.6)	907 (42.8)	<0.001
CV Disease, *n* (%)	213 (3.1)	354 (5.2)	<0.001	18 (0.8)	36 (1.7)	0.020
Hypertension, *n* (%)	900 (13.1)	1744 (25.4)	<0.001	172 (7.9)	290 (13.3)	<0.001
Metabolic Syndrome, *n* (%)	260 (4.3)	1081 (16.6)	<0.001	114 (7.2)	304 (16.9)	<0.001
Gestational DM, *n* (%)	35 (0.5)	116 (1.7)	<0.001	56 (2.6)	100 (4.6)	<0.001
**Laboratory**						
Triglycerides, mg/dL	103.03 (50.41)	129.56 (66.74)	<0.001	101.05 (50.78)	120.21 (66.20)	<0.001
HDL, mg/dL	57.92 (12.81)	53.66 (12.88)	<0.001	50.93 (11.17)	47.95 (10.54)	<0.001
Triglycerides/HDL	1.92 (1.20)	2.65 (1.80)	<0.001	2.16 (1.43)	2.73 (1.87)	<0.001
Glucose, mg/dL	85.01 (8.58)	104.20 (8.44)	<0.001	83.63 (9.22)	104.87 (9.58)	<0.001
Creatinine, mg/dL	0.68 (0.17)	0.68 (0.15)	0.087	0.56 (0.12)	0.60 (0.45)	0.003
**Drugs**						
Statins, *n* (%)	979 (14.2)	1766 (25.7)	<0.001	131 (6.0)	281 (12.9)	<0.001
Thiazide Diuretics, *n* (%)	253 (3.7)	565 (8.2)	<0.001	36 (1.7)	85 (3.9)	<0.001

Continuous variables are presented as mean (SD) unless otherwise stated; categorical variables as *n* (%).

**Table 3:** Multivariate Logistic Regression Analysis.

	Males		Females	
Clinical Factors	Jewish	Bedouin	Jewish	Bedouin
Metabolic syndrome	2.9 (2.4–3.4) *	2.0 (1.4–2.7) *	4.0 (3.5–4.6) *	2.5 (2.0–3.2) *
Cardiovascular disease	1.7 (1.4–2.1) *	2.4 (1.4–4.3) *	1.2 (1.0–1.5) *	1.6 (0.9–2.9)
Gestational diabetes	—	—	3.6 (2.4–5.5) *	2.0 (1.4–2.9) *
Statins	2.1 (1.8–2.4) *	2.3 (1.7–3.0) *	2.0 (1.8–2.2) *	2.1 (1.7–2.7) *
Thiazide diuretics	1.4 (1.1–1.9) *	2.6 (1.2–6.2) *	1.5 (1.3–1.8) *	1.6 (1.0–2.4) *

Adjusted odds ratios (OR) and 95% confidence intervals from multivariable logistic regression analysis. * Statistically significant association.

## Data Availability

Data available on request due to restrictions.

## Supplementary Materials

The following supporting information can be downloaded at: https://www.mdpi.com/article/10.3390/jcm15134893/s1, Table S1: Bootstrap-derived adjusted odds ratios and 95% confidence intervals based on 500 bootstrap replications of the multivariable logistic regression models. Figure S1: Expanded multivariate model illustrating the contributions of individual components of metabolic syndrome to prediabetes risk across subgroups.

## Abbreviations

American Diabetes Association	ADA
Impaired fasting glucose	IFG
Impaired glucose tolerance	IGT
Oral glucose tolerance test	OGTT
Clalit Health Services	CHS
Health maintenance organization	HMO
Indonesian Prediabetes Risk Score	INA-PRISC
U.S National Health and Nutrition Examination Survey	NHANES
Ischemic heart disease	IHD
Hypertension	HTN
Peripheral vascular disease	PVD
Gestational diabetes mellitus	GDM
Cerebrovascular accident	CVA
Transient ischemic attack	TIA
Acetylsalicylic acid	ASA
Statins	STA
Thiazide diuretics	TZD
Cardiovascular disease	CVD
Standard deviations	SDs
Triglyceride to HDL cholesterol ratio	TG/HDL
Odds ratio	OR
Finnish Diabetes Risk Score	FINDRISC
Diabetes Prevention Program and its Outcomes Study	DPPOS
