# Supplementary material for: Ethnic and Gender Disparities in Risk Factors for Prediabetes—A Retrospective Exploratory Analysis in Southern Israel
Source: J Clin Med. 2026 Jun 23;15(13):4893. doi: 10.3390/jcm15134893 (PMC13361226; doi:10.3390/jcm15134893)
Supplement: Supplementary file 1 [file jcm-15-04893-s001.zip › jcm-4319440-supplementary.pdf]

Supplementary Table S1

Supplementary Table S1. Bootstrap-derived adjusted odds ratios and 95% confidence intervals based on 500 bootstrap replications of the multivariable logistic regression models

Variable	Jewish Male	Arab Male	Jewish Female	Arab Female
Metabolic syndrome	2.87 (2.42–3.40)	1.97 (1.44–2.74)	4.01 (3.52–4.69)	2.54 (1.99–3.17)
CVD_Total	1.70 (1.38–2.06)	2.38 (1.38–4.42)	1.22 (0.99–1.49)	1.56 (0.88–3.11)
Gestational diabetes	—	—	3.56 (2.39–5.57)	1.95 (1.41–2.88)
Statins	2.05 (1.77–2.32)	2.29 (1.79–2.97)	1.99 (1.79–2.19)	2.13 (1.70–2.70)
Low-ceiling (thiazide) diuretics	1.43 (1.11–1.93)	2.58 (1.24–7.15)	1.51 (1.29–1.79)	1.57 (1.02–2.45)

Values are adjusted odds ratios with bootstrap 95% confidence intervals. Em dashes (—) indicate variables not applicable to the corresponding stratum

Supplementary figure S1

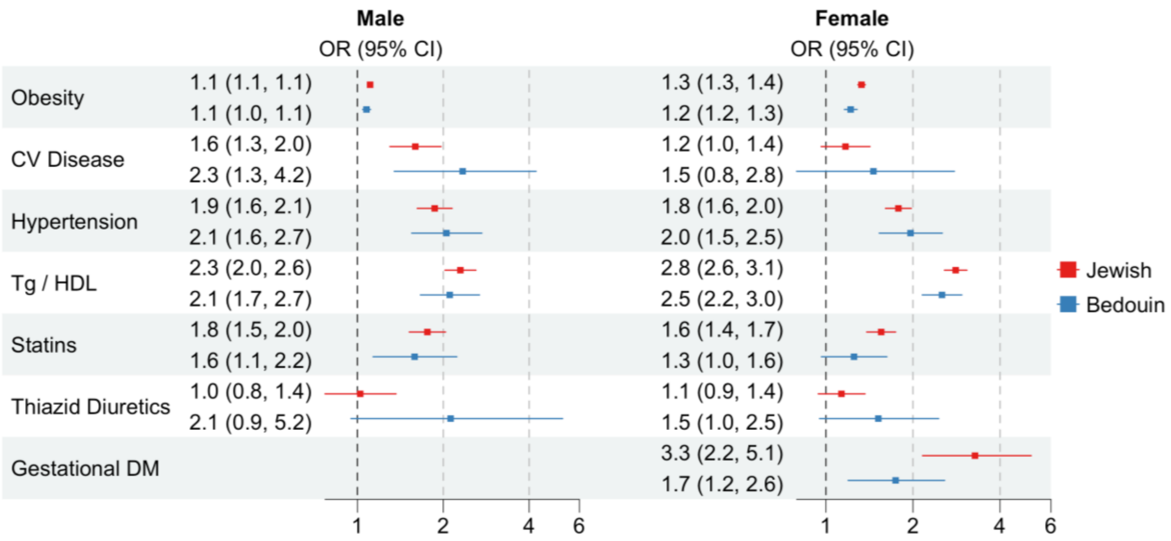

The components include body mass index (BMI), triglycerides (TG), high-density lipoprotein cholesterol (HDL), hypertension (HTN), and fasting glucose. Obesity (BMI >30 kg/m²) was identified as the most prominent contributor across all ethnic and gender groups. This figure underscores the central role of metabolic syndrome and its components in prediabetes development
